# Colistin- and Carbapenem-Resistant *Escherichia coli* Harboring *mcr-1* and *bla*_NDM-5_, Causing a Complicated Urinary Tract Infection in a Patient from the United States

**DOI:** 10.1128/mBio.01191-16

**Published:** 2016-08-30

**Authors:** José R. Mediavilla, Amee Patrawalla, Liang Chen, Kalyan D. Chavda, Barun Mathema, Christopher Vinnard, Lisa L. Dever, Barry N. Kreiswirth

**Affiliations:** a Public Health Research Institute Tuberculosis Center, New Jersey Medical School, Rutgers University, Newark, New Jersey, USA; b Division of Pulmonary and Critical Care Medicine, New Jersey Medical School, Rutgers University, Newark, New Jersey, USA; c Department of Epidemiology, Mailman School of Public Health, Columbia University, New York, New York, USA; d Division of Infectious Diseases, New Jersey Medical School, Rutgers University, Newark, New Jersey, USA

## Abstract

Colistin is increasingly used as an antibiotic of last resort for the treatment of carbapenem-resistant Gram-negative infections. The plasmid-borne colistin resistance gene *mcr-1* was initially identified in animal and clinical samples from China and subsequently reported worldwide, including in the United States. Of particular concern is the spread of *mcr-1* into carbapenem-resistant bacteria, thereby creating strains that approach pan-resistance. While several reports of *mcr-1* have involved carbapenem-resistant strains, no such isolates have been described in the United States. Here, we report the isolation and identification of an *Escherichia coli* strain harboring both *mcr-1* and carbapenemase gene *bla*_NDM-5_ from a urine sample in a patient without recent travel outside the United States. The isolate exhibited resistance to both colistin and carbapenems, but was susceptible to amikacin, aztreonam, gentamicin, nitrofurantoin, tigecycline, and trimethoprim-sulfamethoxazole. The *mcr-1*- and *bla*_NDM-5_-harboring plasmids were completely sequenced and shown to be highly similar to plasmids previously reported from China. The strain in this report was first isolated in August 2014, highlighting an earlier presence of *mcr-1* within the United States than previously recognized.

## IMPORTANCE

Colistin has become the last line of defense for the treatment of infections caused by Gram-negative bacteria resistant to multiple classes of antibiotics, in particular carbapenem-resistant *Enterobacteriaceae* (CRE). Resistance to colistin, encoded by the plasmid-borne gene *mcr-1*, was first identified in animal and clinical samples from China in November 2015 and has subsequently been reported from numerous other countries. In April 2016, *mcr-1* was identified in a carbapenem-susceptible *Escherichia coli* strain from a clinical sample in the United States, followed by a second report from a carbapenem-susceptible *E. coli* strain originally isolated in May 2015. We report the isolation and identification of an *E. coli* strain harboring both colistin (*mcr-1*) and carbapenem (*bla*_NDM-5_) resistance genes, originally isolated in August 2014 from urine of a patient with recurrent urinary tract infections. To our knowledge, this is the first report in the United States of a clinical bacterial isolate with both colistin and carbapenem resistance, highlighting the importance of active surveillance efforts for colistin- and carbapenem-resistant organisms.

## Observation

Polymyxins are increasingly used as antibiotics of last resort for clinical infections caused by carbapenem-resistant Gram-negative bacteria, especially carbapenem-resistant *Enterobacteriaceae* (CRE) (1). The recent discovery of transmissible colistin resistance in China (2) has led to numerous reports of the plasmid-borne gene *mcr-1* in animal, food, and clinical samples worldwide (3). Of particular concern is the spread of *mcr-1* into CRE, thereby creating highly drug-resistant strains that are potentially untreatable. Several reports outside the United States have already described *mcr-1* in bacterial strains harboring carbapenemases, including NDM-1 (4), NDM-5 (5–7), NDM-9 (8), KPC-2 (9), OXA-48 (10), and VIM-1 (11). Most recently, *mcr-1* was detected in two clinical carbapenem-susceptible *Escherichia coli* strains from the United States, isolated from urine cultures obtained in April 2016 (12) and May 2015 (13), respectively. Here, we report the identification from urine of an *E. coli* strain harboring both *mcr-1* and carbapenemase gene *bla*_NDM-5_, originally isolated in August 2014 from a U.S. patient with recurrent urinary tract infections and no recent travel history.

A 76-year-old man presented to a tertiary-care hospital in New Jersey with subjective fever and flank pain in August 2014. The patient had emigrated from India and resided continuously in the United States for 1 year prior to this presentation. He had a history of prostate cancer treated with radiation therapy and subsequently developed recurrent urinary tract infections. He had recently undergone cystoscopy to evaluate the possibility of colovesicular fistula formation. The procedure was complicated by bladder perforation requiring bilateral nephrostomy tube placement. The nephrostomy tubes were clamped 5 days prior to presentation, and he then developed subjective fever, chills, and generalized weakness. Laboratory testing on presentation indicated a leukocyte count of 14.1 × 10^3^ cells/µl, associated with pyuria. The initial antimicrobial regimen included intravenous piperacillin-tazobactam and vancomycin. No fistula was seen on imaging studies, and the nephrostomy tubes were unclamped.

The susceptibility results of urine cultures obtained prior to the initiation of antimicrobial therapy are shown in Table 1. A clean-catch urine culture grew greater than 100,000 CFU per ml of *Pseudomonas aeruginosa*, *Citrobacter koseri*, and *Enterococcus faecium*. A urine culture obtained from the nephrostomy tube grew >100,000 CFU/ml of *P. aeruginosa*, *Escherichia coli*, *Klebsiella pneumoniae*, *Enterococcus* spp., and *Staphylococcus aureus* (methicillin resistant). Susceptibility testing results showed that the *E. coli* isolate was resistant to colistin and all β-lactams (including carbapenems) except aztreonam, but remained susceptible to amikacin, gentamicin, nitrofurantoin, tigecycline, and trimethoprim-sulfamethoxazole (Table 1). The *P. aeruginosa* isolate was susceptible to piperacillin-tazobactam and colistin but exhibited resistance or intermediate resistance to other agents tested, including carbapenems. In contrast, the *C. koseri* and *K. pneumoniae* isolates were resistant to only ampicillin, with the latter also exhibiting intermediate resistance to nitrofurantoin. Based on these culture results, oral trimethoprim-sulfamethoxazole was added to the antimicrobial regimen, although by this point the patient had remained afebrile and the leukocyte count had normalized. A sterile clean-catch urine culture was obtained after 6 days of antimicrobial therapy. The patient returned home after undergoing a urinary diversion with ileal conduit.

**TABLE 1:** Antimicrobial susceptibilities of Gram-negative bacterial species isolated from urine cultures and nephrostomy tube drainage obtained from the case patient^e^

Antimicrobial agent^a^	*E. coli*^b^		*C. koseri*^c^		*K. pneumoniae*^b^		*P. aeruginosa*^b^^,^^c^	
	MIC (μg/ml)	Interp	MIC (μg/ml)	Interp	MIC (μg/ml)	Interp	MIC (μg/ml)	Interp
Amikacin	≤16	S	≤16	S	≤16	S	>32	R
Ampicillin	>16	R	>16	R	>16	R	N/R	
Ampicillin-sulbactam	>16/8	R	≤8/4	S	≤8/4	S	N/R	
Aztreonam	≤8	S	≤8	S	≤8	S	16	I
Cefazolin	>16	R	≤8	S	≤8	S	N/R	
Cefepime	>16	R	≤8	S	≤8	S	>16	R
Ceftazidime	>16	R	≤1	S	≤1	S	>16	R
Ciprofloxacin	>2	R	≤1	S	≤1	S	>2	R
Colistin	3	R^d^	N/R		N/R		2	S^d^
Ertapenem	>4	R	≤1	S	≤1	S	>4	R
Gentamicin	≤4	S	≤4	S	≤4	S	>8	R
Imipenem	>8	R	≤4	S	≤4	S	>8	R
Levofloxacin	>4	R	≤2	S	≤2	S	>4	R
Meropenem	>8	R	≤4	S	≤4	S	>8	R
Nitrofurantoin	≤32	S	≤32	S	64	I	>64	R
Piperacillin-tazobactam	>64	R	≤16	S	≤16	S	64	S
Tigecycline	≤2	S	≤2	S	≤2	S	N/R	
Trimethoprim-sulfamethoxazole	≤2/38	S	≤2/38	S	≤2/38	S	N/R	

a Antimicrobial susceptibilities for all agents (except colistin) were obtained using the MicroScan Walkaway Plus System (Beckman Coulter, Brea, CA).

b Isolated from nephrostomy tube drainage culture.

c Isolated from clean-catch urine culture.

d Colistin MIC values were determined using Etest strips (bioMérieux, Marcy-l’Étoile, France). The MICs for *E. coli* and *P. aeruginosa* were 3 µg/ml and 2 µg/ml, respectively.

e Abbreviations: MIC, minimum inhibitory concentration; Interp, interpretation; R, resistant; I, intermediate; S, susceptible; N/R, not reported.

Since 2014, our laboratory has used molecular methods to analyze clinical isolates of Gram-negative bacteria obtained from this affiliated tertiary-care hospital, including 16S sequencing; multilocus sequence typing (MLST); and PCR detection of carbapenemases, AmpC β-lactamases, extended-spectrum β-lactamase genes, and, more recently, the *mcr-1* gene. Using these methods, the *E. coli* isolate from the study case (named MCR1_NJ) was shown to carry both *mcr-1* and *bla*_NDM-5_ genes. Whole-genome sequencing of *E. coli* strain MCR1_NJ was performed using an Illumina NextSeq platform (San Diego, CA), and the resistome was investigated using ResFinder 2.1 (14). In addition to *mcr-1* and *bla*_NDM-5_, strain MCR1_NJ was found to harbor resistance genes for aminoglycosides [*strA*, *strB*, and *aac(6′)-Ib-cr*], β-lactams (*bla*_OXA-1_), chloramphenicol (*catB3* and *floR*), fluoroquinolones [*aac(6′)-Ib-cr*], rifampin (*arr-3*), sulfonamides (*sul1* and *sul2*), and tetracycline [*tet*(A)].

The *mcr-1*- and *bla*_NDM-5_-harboring plasmids from *E. coli* strain MCR1_NJ were transferred to *E. coli* DH10B by electroporation, thereby confirming the mutually exclusive presence of *mcr-1* and *bla*_NDM-5_ in the resulting transformants. The conjugability of the *mcr-1* plasmid was further confirmed by experiments using *E. coli* J53 Azi^r^ as the recipient strain. Plasmid DNA was isolated using a Qiagen Plasmid Midi kit (Hilden, Germany) and subjected to complete plasmid sequencing using Illumina NextSeq as described previously (6). Sequencing reads were assembled *de novo* using SPAdes software (15), and gaps were closed by Sanger sequencing as described previously (6, 16). The *mcr-1*-harboring plasmid from *E. coli* MCR1_NJ (subsequently named pMCR1-NJ-IncX4) was 33,395 bp in length and had 100% BLAST query coverage and 99.6% nucleotide identity to conjugative plasmid pMCR1-IncX4 (GenBank accession no. KU761327), which we previously described in CTX-M-55-producing *E. coli* and NDM-5-producing *K. pneumoniae* strains from Chinese hospitals (5, 6). The *bla*_NDM-5_-harboring plasmid (named pNDM5-NJ-IncX3) was 39,520 bp in length and closely related (100% nucleotide identity and 79% query coverage) to pNDM5-IncX3 (accession no. KU761328), previously described in the two aforementioned NDM-5-producing *K. pneumoniae* strains from China (6). No other resistance genes besides *mcr-1* and *bla*_NDM-5_ were observed in plasmid pMCR1-NJ-IncX4 or pNDM5-NJ-IncX3, respectively.

Strain MCR1_NJ was shown by MLST to be a single-locus variant of ST405, associated with *E. coli* phylogroup D (17). ST405 is classified as one of the main extraintestinal pathogenic *E. coli* (ExPEC) lineages (18), and is associated with the global spread of extended-spectrum β-lactamases, most notably CTX-M-15 (18–20). AmpC cephamycinases and NDM metallo-β-lactamases have also been reported in ST405 (20), including NDM-1 (21) and NDM-4 (22). Consequently, ST405 may also be involved in the global dissemination of NDM-producing *E. coli* strains (22). Moreover, whereas strain MCR1_NJ was susceptible to various antibiotics, including gentamicin and trimethoprim-sulfamethoxazole, studies of ST405 strains from multiple countries suggest that they are typically resistant to these as well (20). Worrisomely, ST405 has been frequently associated with community onset urinary tract infections (23–25). Dissemination of *mcr-1* within this global lineage may therefore contribute to further spread of polymyxin resistance within ESBL- and carbapenemase-producing *E. coli* (and other *Enterobacteriaceae*) strains.

In summary, we report the isolation and identification of an *E. coli* strain harboring both *mcr-1* and *bla*_NDM-5_ from urine of a patient without recent travel outside the United States. This strain was isolated in August 2014, highlighting an earlier presence of *mcr-1* within the region than previously known and raising the likelihood of ongoing undetected transmission. Active surveillance efforts involving all polymyxin- and carbapenem-resistant organisms are imperative in order to determine *mcr-1* prevalence and prevent further dissemination.

### Accession number(s).

The complete nucleotide sequences of plasmids pMCR1-NJ-IncX4 and pNDM5-NJ-IncX3 have been deposited as GenBank accession no. KX447768 and KX447767, respectively. The draft genome sequence of *E. coli* strain MCR1_NJ was deposited as GenBank accession no. MAJK00000000.
